# Remodeling Components of the Tumor Microenvironment to Enhance Cancer Therapy

**DOI:** 10.3389/fonc.2015.00214

**Published:** 2015-10-14

**Authors:** Vasiliki Gkretsi, Andreas Stylianou, Panagiotis Papageorgis, Christiana Polydorou, Triantafyllos Stylianopoulos

**Affiliations:** ^1^Cancer Biophysics Laboratory, Department of Mechanical and Manufacturing Engineering, University of Cyprus, Nicosia, Cyprus; ^2^Program in Biological Sciences, Department of Health Sciences, European University Cyprus, Nicosia, Cyprus

**Keywords:** tumor vessel permeability, vessel compression, vascular normalization, stress alleviation, *in vitro* models

## Abstract

Solid tumor pathophysiology is characterized by an abnormal microenvironment that guides tumor progression and poses barriers to the efficacy of cancer therapies. Most common among tumor types are abnormalities in the structure of the tumor vasculature and stroma. Remodeling the tumor microenvironment with the aim to normalize any aberrant properties has the potential to improve therapy. In this review, we discuss structural abnormalities of the tumor microenvironment and summarize the therapeutic strategies that have been developed to normalize tumors as well as their potential to enhance therapy. Finally, we present different *in vitro* models that have been developed to analyze and better understand the effects of the tumor microenvironment on cancer cell behavior.

## Introduction

Tumors have long been considered as complex tissues in which mutant cancer cells have summoned normal cell types that serve as active collaborators toward a neoplastic phenotype. Thus, malignant cancer cells, despite all accumulated mutations, do not act alone in cancer progression. Hence, the interactions and crosstalk between malignant cells and the supporting cell types that form the tumor microenvironment are critical for better understanding of cancer pathogenesis as well as for the development of novel and more effective therapies (1).

Structural components of the tumor microenvironment are the tumor blood and lymphatic vessels, the extracellular matrix (ECM) with most common constituents being collagen and hyaluronic acid, and the stromal cell constituents of the tumor. According to a recent review by Hanahan and Coussens (2), the latter can be divided into three categories: (a) angiogenic vascular cells, which include endothelial cells, and pericytes, (b) infiltrating immune cells, which include platelets, mast cells, neutrophils, inflammatory monocytes, myeloid-derived suppressor cells (MDSCs) (3), macrophages (4), CD8 T-cells, NK T-cells, CD4 T-cells (5), and B cells, and (c) cancer-associated fibroblastic cells (CAFs), which include activated tissue fibroblasts, activated adipocytes, a-smooth muscle actin (a-SMA) + myofibroblasts and mesenchymal stem cells (MSCs). Importantly, the above-mentioned components of tumor microenvironment may vary depending on the type of the tumor and its location, making each tumor unique.

The effects of tumor microenvironment on cancer cell properties, including proliferation, apoptosis, migration, and invasion, are pleiotropic and determined by both direct and indirect interactions of cancer cells with components of their microenvironment.

Apart from regulating the behavior of cancer cells, abnormalities of the tumor vasculature and stroma pose barriers to the effective delivery of therapeutic agents, which can result in compromised treatment outcomes. Indeed, in many tumor types, such as pancreatic ductal adenocarcinomas (PDACs) and subsets of breast tumors and sarcomas, these barriers can become insurmountable, often leading to therapy failure (6).

### Abnormalities of tumor vasculature and effects on tumor progression

A well-studied abnormality of the tumor microenvironment is the hyperpermeability of the tumor blood vessels (7, 8). Pro-angiogenic factors that induce angiogenesis [e.g., vascular endothelial growth factor (VEGF), platelet-derived growth factor (PDGF)] are upregulated in most tumors and can drive the formation of immature vessels with structural abnormalities. In these vessels, the endothelial lining that forms the vessel wall can have wide junctions, large numbers of fenestrae and intercellular openings, and be accompanied by a disorganized or loose basement membrane and incomplete pericyte coverage (9). As a result, whereas in normal vessels the pore cut off size of the vessel wall is usually less than 12 nm in diameter (10), in tumor vessels it can be up to two orders of magnitude larger (11, 12).

Despite the fact that not all tumor vessels are hyperpermeable and they might be heterogeneously distributed inside the tumor, vessel hyperpermeability is a hallmark of tumor pathophysiology and one of its consequences is a reduction in tumor perfusion (i.e., blood supply) owing to excessive fluid loss from the vascular to the extravascular space of the tumor (6, 13). Hypo-perfusion, in turn, can result in hypoxia and formation of a harsh, acidic microenvironment that fuels tumor progression. Indeed, reduced perfusion prevents cells of the immune system to reach the tumor site through the vascular network. Immune cells patrol the human body to eliminate pathogens, foreign antigens, and abnormal cells and, thus, they need an effective vascular system to be efficient (14). Furthermore, hypoxia and low pH attenuate the killing potential of immune cells, such as T lymphocytes and dendritic cells, reduce their proliferation rate and reprogram macrophages into an immunosuppresive, pro-tumorigenic phenotype (2, 15–19). Hypoxia and acidosis can also promote a more malignant phenotype, since only cells that are resistant to these extreme conditions will be able to survive. Furthermore, such environment can enhance the proliferation rate of cancer stem cells and induce a more invasive and metastatic phenotype (20). Finally, reduced blood supply can drastically decrease the accumulation of chemotherapeutic and nanotherapeutic agents into the tumor interior while hypoxia itself is known to compromise the efficacy of radiation therapy (6, 14).

Apart from reducing vascular blood flow, excessive fluid loss owing to tumor vessel hyperpermeability causes a uniform elevation of the fluid pressure of the tumor, known as interstitial fluid pressure (21, 22). Fluid communication between the vascular and extravascular space results in an equilibrium when the vascular and interstitial pressures are comparable. Equalization of fluid pressures across the tumor vessel wall poses a major barrier to the extravasation of therapeutics because there is no fluid flow to drive their transport inside the tumor. Indeed, the main mechanism of transport is diffusion, which is a size-dependent mechanism. Therefore, the larger the drug the more difficult it will enter the tumor, which primarily affects the delivery of nanomedicines (23, 24). Fluid pressure can be uniform not only across the vessel wall but also in the interior of the tumor, rendering diffusion a dominant transport mechanism for intratumoral transport, thus inhibiting the penetration and homogeneous distribution of nanoparticles (25). Finally, interstitial fluid pressure drops to normal values at the periphery of the tumor where lymphatics are functional and hyper-plastic. The steep decrease of the fluid pressure at the tumor margin creates a fluid flux outward of the tumor, which can drift tumor-generated growth factors, therapeutic agents, and metastatic cancer cells to the surrounding normal tissues promoting tumor progression (26).

Another, less studied, abnormality of the tumor vasculature is the compression of intratumoral blood and lymphatic vessels. Vessel compression is a result of mechanical forces accumulated within the tumor during progression owing to abnormalities in the tumor stroma (27–29). Blood vessel compression contributes significantly to tumor hypo-perfusion since an upstream compressed vessel can exclude a large number of downstream vessels from blood supply. Therefore, tumor blood vessel compression contributes to the processes associated with hypo-perfusion, hypoxia, and acidosis discussed previously. Finally, compression of lymphatics hinders the capacity of the tumor to drain the excessive fluid that enters the tumor through the hyperpermeable blood vessels, to further cause elevation of the interstitial fluid pressure.

### Abnormalities of tumor ECM and effects on tumor progression

Extracellular matrix is a fundamental component of the tumor microenvironment that acts bidirectionally, both affecting and being affected by tumor cells. Through interactions with the integrins receptors, ECM communicates with the interior of the cell accepting and transmitting survival signals (30). Besides providing cell-adhesion sites, ECM is also critical for tissue homeostasis as a reservoir for growth factors. In fact, many growth factors have the ability to bind specific sites within the ECM, leading to the release of signaling molecules at different kinetics and from different locations, allowing a well-orchestrated regulation of cell fate within the tumor microenvironment (31). Moreover, remodeling the ECM through the secretion of matrix metalloproteinases (MMPs) from CAFs and/or tumor cells facilitates the release of bound-to-ECM growth factors, thus promoting tumor growth.

Interestingly, another connection has been reported between the ECM and cancer development. More specifically, it has been long known that cancer bears many similarities to unhealed wounds, leading many researchers to investigate wound healing as a means to better understanding cancer and/or discovering new therapeutic interventions for the disease (31–33). Indeed, malignant tumors often develop at sites of chronic injury, while the healing of a wound by itself with the ECM accumulation and the formation of the scar greatly resembles the development of a tumor apart from the invasive capacity that is not present in cells participating in wound healing.

The abnormal structure and function of tumor stroma is largely attributed to the upregulation of matrix remodeling molecules such as the transforming growth factor β (TGFβ) (34). These abnormalities, collectively known as *desmoplasia*, refer to the formation of a dense ECM characterized by increased levels of total fibrillar collagen, fibronectin, proteoglycans (PGs), and tenascin C. In fact, desmoplastic response has been demonstrated to be associated with more aggressive and invasive cancer and worse prognosis in several types of cancer such as papillary thyroid microcarcinomas (35), breast cancers (36), and rectal cancers (37).

Furthermore, a desmoplastic response involves the transformation of fibroblasts to myofibroblasts or CAFs. CAFs are large, spindle-shaped mesenchymal cells that share characteristics with smooth muscle cells and fibroblasts (38). They constitute a significant stromal component and represent the cells responsible for the change of ECM composition toward desmoplasia, characterized by increased amounts of collagen deposition. Although CAFs are both phenotypically and functionally distinct from their normal counterparts and they are identified immunohistochemically by different markers, such as α-SMA, vimentin, desmin, fibroblast-specific protein-1, and fibroblast activation protein, there is hardly any true definition of CAFs (39). Researchers agree, though, that CAFs can promote tumor progression in a multitude of ways, such as secretion of multiple growth factors and MMPs, secretion of factors that induce stemness, or epithelial-to-mesenchymal transition (EMT) (39).

Desmoplastic response and thus ECM deposition differs significantly among various cancer types, indicating different mechanisms of cancer development and progression. Moreover, in certain cases, ECM components show different localization patterns even within the same tumor depending on the differentiation and the histological pattern of tumors (40). Thus, although, knowing the exact composition of ECM in each cancer type would be useful and perhaps clinically and/or diagnostically relevant, current literature lacks extensive studies of ECM deposition in different human cancers. In the present review, we summarize existing studies of ECM deposition in human samples of breast, colon, lung cancer, pancreatic cancer, hepatocellular carcinoma, and glioblastoma (Table 1).

**Table 1:** **Tumor-specific ECM deposition for common types of cancer**.

Type of cancer	Tumor-specific ECM deposition	Reference
Breast cancer	Collagen I, collagen IV, collagen V, fibronectin, laminins, entactin, proteoglycans, glycosaminoglycans	(41)
Colon cancer	Chondroitin sulfate proteoglycan, hyaluronic acid Laminin, collagen IV, heparan sulfate proteoglycan	(42, 43)
Glioblastoma	Collagen IV, procollagen III, laminin, fibronectin, and hyaluronic acid Fibrillar collagens (i.e., collagens I, III)	(44, 45)
Hepatocellular carcinoma	Collagen IV and laminin	(46)
Lung cancer	Collagen types I and III, non-collagenous glycoproteins laminin, and fibronectin	(47)
Pancreatic cancer	Collagen I, hyaluronan Collagens I, III, and IV, laminin, tenascin, vitronectin	(48) (49)

Tumor types known to be highly desmoplastic include subsets of pancreatic and breast cancers as well as sarcomas. Desmoplasia may contribute to an increase in tumor density which, as a tumor grows in the confined space of the host tissue, results in the generation and accumulation of mechanical forces among the components of the tumor microenvironment as well as between the tumor and the host tissue (50). Indeed, stromal cells and extracellular components, predominantly collagen and hyaluronic acid, exert forces on tumor blood and lymphatic vessels causing their compression and eventually their collapse. Stromal cells and ECM exert forces not only on tumor vessels but also on cancer cells. Compression of cancer cells has been found to reduce their proliferation rate, induce apoptosis, and promote a more invasive and metastatic phenotype (51–55). Therefore, mechanical forces affect tumor progression both directly, by applied to cancer cells, and indirectly, via intratumoral vessel compression.

Finally, desmoplasia limits the available space for transport in the tumor interstitial space, which prevents homogeneous penetration of therapeutic agents (9, 56). High collagen and cellular densities reduce the size of the pores of the tumor interstitial space and as a result the resistance to interstitial fluid flow increases. This, in turn, further enhances the uniform elevation of the interstitial fluid pressure and renders diffusion the dominant transport mechanism in the tumor interior. Therefore, when therapeutics, particularly particles large in size (>60 nm), extravasate from the hyperpermeable tumor vessels, might not be able to effectively penetrate deep into the tumor either because they are trapped due to their size being comparable or larger than the size of the pores of the interstitial space or because, by definition, diffusion is a slow transport mechanism for large nanoparticles. As a consequence, the nanoparticles will be concentrated in the perivascular regions resulting in heterogeneous drug distribution which primarily causes local effects (57, 58).

## Targeting Molecules That Modulate the Tumor Microenvironment

The conventional assumption is that multiple mutations in cancer cells after prolonged exposure to therapeutic agents can result in their drug-resistant clonal expansion leading to disease progression and eventually patient death (59). However, it is generally realized over the last decades that this mechanism represents only one of the underlying causes for this phenomenon. It is now becoming increasingly clear that the tumor microenvironment can also play crucial roles both in promoting tumor progression and in determining the efficacy of cancer therapy. To this end, research efforts have recently revealed several promising target molecules (60).

As it has already been mentioned, abnormal tumor vasculature is a hallmark of most cancers and is often the result of the imbalance between pro- and anti-angiogenic signaling in cancer or stromal cells (61). Angiogenesis is a process that is largely driven by VEGF, which can be derived from various sources. Increased levels of the major pro-angiogenic factor VEGF and its receptors can be produced by cancer cells and have been associated with resistance to chemotherapy in a variety of human tumor models, including colorectal, gastric, sarcoma, and pancreatic cancers (62–65). VEGF expression is transcriptionally induced under hypoxic conditions that stabilize the hypoxia-inducible factors 1 and 2 (HIF-1α and HIF-2α) by reducing the activity of the prolyl hydroxylase domain proteins 1–3 (PHD1–3) (66). Importantly, CAFs can also promote angiogenesis and drug resistance by secreting numerous pro-angiogenic molecules, including VEGF (67), stromal cell-derived factor 1 (SDF-1) (68), platelet-derived growth factors B and C (PDGF-B,C) (69), fibroblast growth factors 2 and 7 (FGF-2 and FGF-7) (70). In addition, vessel maturation and stabilization is controlled by molecules such as PDGF-B, regulator of G-protein signaling 5 (RGS5), angiopoietin-1/2 (Ang1/2), and TGFβ by recruiting perivascular cells (pericytes and vascular smooth muscle cells) (61, 71).

Besides its important functions in the physiological tissue growth, differentiation, and homeostasis, the ECM also plays crucial roles during cancer progression. ECM is a highly heterogeneous and dynamic structure that interacts with different cellular components and the individual cellular microenvironment of each tissue and constantly becomes remodeled in response to different biomechanical stimuli. It is localized either in the basement membrane or in the interstitial space and is unique for each tissue. Although it is mainly composed of water, proteins, and polysaccharides, there are two classes of macromolecules that can be identified: PGs and fibrous proteins. All PGs consist of glycosaminoglycan (GAG) chains that are covalently linked to a protein core molecule, with the exception of the highly abundant hyaluronic acid. PGs are classified according to their core proteins, localization, and GAG composition into three main families: small leucine-rich proteoglycans (SLRPs), modular PGs, and cell-surface PGs (72, 73) and include members such as aggrecan, decorin, glypican, and syndecan (73). The major fibrous ECM proteins include different types of collagens, elastins, laminins, tenascin, and fibronectin while PGs occupy the majority of the remaining extracellular space (74). Although to date 28 different types of collagen have been identified (72, 73), collagens I, III, and IV are the most highly represented proteins in the ECM and exert important functions in regulating tissue development, tensile strength, cell adhesion, and migration (75). The majority of collagen is synthesized and secreted by fibroblasts and CAFs in response to TGFβ stimulation and local application of mechanical forces (34). Its structure is characterized by triple-stranded helices that are assembled together with the help of different TGFβ-regulated crosslinking enzymes, such as lysyl oxidase (LOX), connective tissue growth factor (CTGF), and periostin (POSTN) (76–78). Finally, fluctuations in the activity of MMPs may also have a great impact on ECM remodeling (79).

It should be mentioned, however, that commonly used cytotoxic therapies such as radiation and chemotherapy tend to remodel the tumor stroma by themselves, by activating several of its components and promoting tumor growth (80–82). For instance, it has been shown that exposure of CAFs isolated from human lung tumors to ablative ionizing radiation mediates a transformation on their secretory profile that includes downregulation of angiogenic molecules and upregulation of FGF, all of which can certainly affect cell behavior within the tumor and thus guide therapeutic outcomes (81). Moreover, ionizing radiation was shown to induce rapid and global changes in the mammary microenvironment characterized by altered ECM composition and growth factor activities (80), reinforcing the idea that radiation-induced changes in the stromal microenvironment can contribute to neoplastic progression *in vivo*. Finally, chemotherapy-treated human CAFs were shown to promote colorectal cancer initializing cells self-renewal and *in vivo* tumor growth (82), indicating that chemotherapy also induces remodeling of the tumor microenvironment.

To counteract this effect that chemotherapy and radiation may have on tumors and tumor microenvironment, combination therapy is usually undertaken to improve clinical outcome (83). Interestingly, a number of preclinical and clinical studies have shown that radiation and chemotherapy elicit changes within tumors and their microenvironment that make the malignant cells more sensitive to an efficient immune cell attack, thus suggesting that a combination of immunotherapy with standard anticancer therapies will provide synergistic antitumor effects (84, 85). Last but not least, a combination of chemotherapy and/or radiotherapy with anti-angiogenic agents has also been suggested as suitable for better outcomes as these agents target tumor vasculature and new tumor vessel formation and can modulate the tumor microenvironment to improve tumor blood flow and oxygenation, leading to enhanced radio- and chemo-sensitivity (83).

## Therapeutic Strategies to Remodel the Tumor Microenvironment and Enhance Therapy

**Figure 1:**
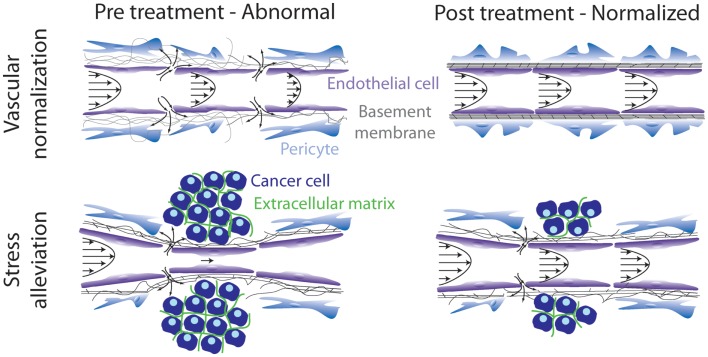
**Schematic of strategies to remodel the tumor microenvironment to enhance cancer therapy**. Vascular normalization treatment fortifies the hyperpermeable tumor blood vessels, whereas stress alleviation reopens compressed tumor blood vessels. Both strategies aim at improving tumor perfusion. Adapted with permission from Ref. (86).

According to our analysis, therapeutic strategies aiming at remodeling the tumor microenvironment to enhance drug delivery can target either the abnormal tumor vessels or the abnormal tumor stroma or both. The basic idea behind these strategies is to bring the tumor to a more “normal” state, i.e., to normalize the tumor vasculature and/or the stroma. Vascular normalization is achieved with judicious doses of anti-angiogenic treatment, targeting mainly the VEGF or its receptors, whereas stroma normalization can be achieved by targeting stroma remodeling molecules, such as TGFβ. In the first case, vascular normalization will restore tumor perfusion by fortifying the vessel wall, which will also drop interstitial fluid pressure due to the reduced amount of fluid leaking from the vessels. In the second case, stroma normalization will improve perfusion by decompressing blood and lymphatic tumor vessels via alleviation of intratumoral mechanical forces (Figure 1).

### Vascular normalization strategy

Anti-angiogenic treatment to prune the tumor vasculature and thus exclude tumors by oxygen and nutrient supply was first introduced by the late Dr. Judah Folkman, more than three decades ago (87). Anti-angiogenic treatment worked well in many preclinical models but failed to improve overall survival when used as monotherapy in most cases in the clinic (88). One of the reasons could be the induction of hypoxia and low pH, which drives tumor progression, as it has been already discussed in this review. In 2001, Jain suggested the use of judicious doses of anti-angiogenic treatment, aiming to normalize the structure and function of tumor vessels, along with cytotoxic agents that will eradicate cancer cells (89). Since then this combinatorial treatment has been employed extensively both in preclinical and in clinical studies with varying degrees of success (14, 61). More specifically, addition of anti-VEGF antibody therapy to standard chemotherapies has improved survival and is an accepted standard of care for a number of cancer types such as cervical cancer (90), colorectal cancer, prostate cancer (91), and advanced non-small cell lung cancer (92). Interestingly though evidence in non-small cell lung cancer also point towards the idea that excessively decreasing vascular permeability and pruning after anti-VEGF therapy may negatively impact the outcome of combination therapy in patients and better treatments are achieved when anti-VEGF treatment results in improved tumor perfusion (92). Moreover, in glioblastoma, which is a highly vascularized type of cancer deemed ideal target for anti-VEGF therapy, it was shown that anti-VEGF therapy improves progression-free survival in newly diagnosed and recurrent glioblastoma, while it was recently suggested that continuing anti-VEGF treatment through multiple lines of therapy might prove beneficial to the patients (93). Furthermore, as in non-small cell lung cancers, vascular normalization improved the efficacy of radiotherapy in glioblastoma patients who exhibited improved tumor perfusion following anti-VEGF treatment, while overall survival was reduced for patients who exhibited hypo-perfusion (94, 95).

The most common targeted molecule in anti-angiogenic treatments has been VEGF (e.g., drug bevacizumab against VEGF-A) and its receptors [e.g., drug cediranib, an inhibitor of all three vascular endothelial growth factor receptors 1–3 (VEGFR-1–3)]. As discussed earlier, a number of other molecular targets present in both cancer and host cells, which have the ability to induce vascular normalization have also been investigated, including PDGF-B,C, HIF, PHD2, Ang1/2 [see Ref. (61) for a detailed review].

The first response of vascular normalization is considered to be an increase in pericyte coverage, which fortifies the vessel wall and reduces the size of its pores (96, 97). Reduction of vessel wall pore size decreases the fluid flux from the vascular to the intratumoral space and, thus, improves perfusion and decreases interstitial fluid pressure, which is beneficial for drug delivery (62). Furthermore, vessel normalization can prune immature, dysfunctional blood vessels forming a vascular network with a more normal structure and improved functionality as far as its capacity to carry blood is concerned. Structural and functional normalization are prerequisites for an anti-angiogenic treatment to become effective in improving delivery of therapeutics (98). Benefits of vascular normalization are, however, dose and time dependent. High or multiple doses of anti-angiogenic treatment can cause excessive pruning of the vasculature, reducing perfusion and drug delivery. Apparently, a balance between the two needs to be reached, which leads to the idea of a normalization window – a function of dose and time within which the strategy is effective (14). Furthermore, vascular normalization improves the delivery of drugs in a size-dependent manner. A reduction in the vessel wall pore size can exclude particles larger than 60 nm in size to effectively cross the tumor vessel wall. Therefore, it has been found that only the delivery of particles with sizes less than 60 nm can be benefited (23).

Clinical studies in humans have verified that anti-angiogenic agents can normalize the tumor vasculature and increased tumor blood perfusion has been shown to prolong survival of patients. Agents that have been successfully used include bevacizumab in patients with rectal cancer, cediranib in patients with recurrent or newly diagnosed glioblastomas and sunitinib in patients with metastatic renal-cell carcinomas (94, 95, 99–102).

### Stress alleviation strategy

Apart from cancer cells, components of the tumor microenvironment that contribute to accumulation of mechanical stresses (i.e., force per unit area) in tumors include the stromal cells, collagen fibers, and hyaluronic acid (50). Selective depletion of any of these constituents in preclinical studies has been found to alleviate stress levels, decompress blood vessels, improve perfusion and drug delivery, and, finally, enhance therapeutic outcomes (86).

Pharmacological depletion of stromal cells using an inhibitor of the Hedgehog pathway (Saridegib) managed to improve chemotherapy in murine pancreatic cancers and the overall survival of the animals (103). A few years later, we found that Saridegib acted as a stress alleviating agent resulting in increased blood vessel diameter and tumor perfusion in murine pancreatic cancers (50). However, more recent studies have shown that excessive depletion of stromal cells may accelerate tumor progression (104–106). Targeting of collagen and hyaluronic acid using angiotensin receptor blockers (ARBs) or angiotensin-converting enzyme (ACE) inhibitors, which are widely used as anti-hypertensive drugs, was found to improve delivery of chemotherapeutic agents and nanomedicines in pancreatic and breast tumors via stress alleviation by decreasing stromal expression of TGFβ as well as other fibrosis-inducing signaling molecules (107, 108). Remodeling of the ECM can be also achieved by other agents that inhibit the TGFβ pathway (109). Additionally, depletion of hyaluronic acid when combined with cytotoxics was found to cause stress alleviation and improved the overall survival of mice bearing pancreatic tumors (110–112). These findings have led to a clinical trial at Massachusetts General Hospital in patients with advanced PDAC – a uniformly fatal disease with very poor prognosis (see ClinicalTrials.GOV – trial identifier number NCT01821729). Retrospective clinical studies have also shown that treatment with ARBs and ACE inhibitors may improve survival in patients with pancreatic, lung, and renal cancers (113–115).

## Methods to Observe Changes in the Tumor Microenvironment Following Pharmacological Intervention

Several studies have used *in vivo* animal models in order to identify the expression of different ECM components in solid tumors. Typical examples include collagen and hyaluronic acid since both have been suggested to play major roles in compressing tumor blood vessels and thus limiting perfusion. These roles within the tumor microenvironment are often examined by establishing animal tumor models followed by pharmacological interventions to modify distinct ECM constituents (6, 50, 108). Xenograft or syngeneic animal tumor models are usually generated by orthotopic or ectopic cancer cell implantation or by implantation of a small piece of viable tumor in mice. Meanwhile, pharmacological treatment of mice is usually administered either orally, using gavage needles directly in the digestive tube, or by intraperitoneal injection or by intravenous injection or by retro-orbital injection (23, 108, 109, 116). Once the pharmacological treatment is completed and when the tumor reaches the desired size, the animals are euthanized followed by tumor excision. Tumors are then prepared either for cryo- or formalin-fixed paraffin embedded sectioning followed by immunohistochemistry. Typically, tumors prepared for paraffin embedding are fixed in paraformaldehyde solution, whereas when tumors are preferred to be processed by cryosectioning, incubated at sucrose/phosphate buffered saline (PBS). Then tumors are embedded in an optimal cutting temperature compound. The tissue thickening during sectioning varies and largely depends on the tumor type, on the primary antibody that will be used and also on the type of preferred staining method. There are various commercially available primary antibodies against collagen and hyaluronic acid that can be used for immunofluorescence and immunohistochemistry. Subsequently, following proper microscopic observation of tumor sections, images taken from each sample can be further processed for analysis and quantification using specific software, such as ImageJ and MATLAB (108, 109, 111, 117).

Animal models can also be used for vascular analysis of tumors. On the last day of treatment, animals are injected with biotinylated tomato lectin either via the retro-orbital sinus or intracardiac injection (108, 118). After tumor removal, the samples are prepared for cryosectioning that will be followed by immunohistochemistry. In most studies, the endothelial marker CD31 is commonly used for staining of all blood vessels followed by counterstaining with secondary antibodies against the biotinylated lectin. Perfused vessels are identified as the ones that display co-localization of biotynilated lectin as well as CD31 staining (108). Further analysis and quantification regarding the perfused vessel area fraction, the diameter, the size, the length, and the density of blood vessels can be performed using various software.

An alternative approach to characterize tumor microenvironment components and observe its changes following pharmacological intervention is with *in vivo* (or commonly referred as *intravital*) microscopy (119, 120). Intravital microscopy includes a number of techniques such as single-photon microscopy, multiphoton (MP) microscopy, and optical frequency domain imaging. According to Fukumura et al., in order to perform intravital microscopy, the components that are required include the preparation of the tissue so that to permit optical access, the use of microscope detectable molecular probes, the use of appropriate microscope/detection systems, and the extraction of parameters of interest through computer algorithms and mathematical models (121). Tissue preparation can be performed through: (1) *in situ* preparation (e.g., ear and tail models), (2) exteriorized tissue preparation, and (3) chronic-transparent windows, including dorsal skinfold chambers, mammary fat pad chambers, and cranial windows (121). Intravital microscopy can be used in living animals so that to monitor and image the expression of specific proteins (e.g., second harmonic generation for fibrillar collagen), the protein subsellular locations and the dynamics of cell populations of interest (122). Intravital MP microscopy technique is a powerful tool that has provided unprecedented mechanistic insights into the tumor microenvironment (120, 123). It can be used for observing single tumor cells and their microenvironment and the same organ (from the same animal) at different time points (122). MP has been used to track individual cancer cells *in vivo* which is useful for the evaluation of cancer cell motility *in vivo* and provides new insights regarding the evolution and response to therapies (124, 125). Furthermore, MP microscopy offers possibilities for direct comparisons of multiple tumor cell populations, such as cancer stem cells and non-stem tumor cells (123). On the other hand, optical frequency domain imaging circumvents some of the technical limitations of MP microscopy, it can be used to monitor the tumor microenvironment, and can also be applied for the evaluation of different the treatment strategies (119, 121).

## *In vitro* Models of Tumor Microenvironment

Although preclinical *in vivo* models are desired for most cancer-related research, reaching that point might require a number of promising reproducible experiments involving cancer cells *in vitro*. Establishing appropriate *in vitro* models that can mimic in detail, the tumor microenvironment is a challenge by itself. Traditionally, cancer researchers have relied on coating tissue culture dishes with purified preparations or mixtures of ECM proteins (i.e., collagen, matrigel, fibronectin, gelatin) in order to obtain two-dimensional (2D) cell monolayers (126). Although 2D-culture models cannot fully recapitulate the tumor microenvironment *in vitro*, they possess certain unique characteristics that enable the investigation of specific cellular, molecular, and biochemical properties of cancer and stromal cells.

### *In vitro* models for the study of mechanical interactions in the tumor microenvironment

Little is known regarding the dynamics of mechanical forces developed within a tumor due to elevating stress or due to ECM stiffening, the ways by which these forces affect cancer cell phenotype as well as the molecular pathways involved. To address these issues, several models have been established to study these interactions *in vitro*.

**Figure 2:**
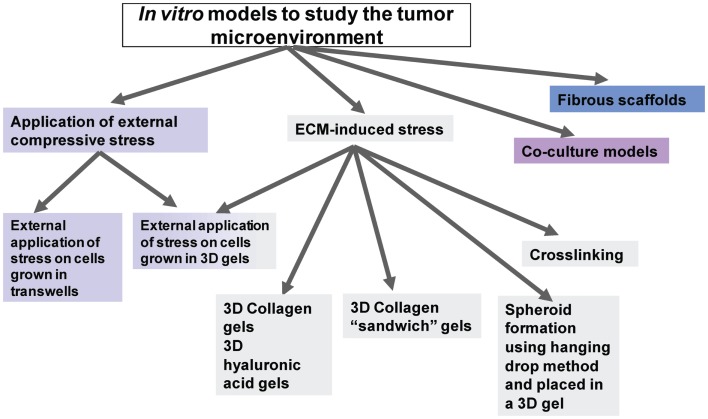
**Diagrammatic representation of the available *in vitro* models to study tumor microenvironment**.

As shown in the diagram of Figure 2, the *in vitro* models used to study the effects of mechanical stress on cancer cell behavior can be divided into two major categories; models in which stress is applied *externally* by means of a mechanical device or other external force, and models in which stress is applied *from within* the tumor microenvironment by means of culturing the cells inside collagen or ECM-containing gels of increasing concentration and, thus, increased stiffness.

#### Externally Applied Stress

##### Using a compression device

In a recent study, novel devices for exerting external compression were developed, namely, a well-pressor and a videomicroscopy-compatible optic-pressor. These devices could exert precise compressive strain (0.25–0.05 kPa for 3 h) on cells embedded in three-dimensional (3D) agarose gels in the absence of other mechanical stimuli and soluble gradients, in aseptic conditions and at physiological temperature and pH conditions. The results from live imaging performed in this study showed that cells elongate and deflect vertically to the load. Moreover, cells tend to differentially regulate the expression of metastasis-associated genes that promote cancer cell invasion (54). Another study used breast cancer cells and “normal” mammary cell lines and subjected them to compressive stress at a level of 5.8 mm Hg using a specially designed *in vitro* compression device. Cell motility was then assessed using the wound healing assay. The authors concluded that compressive stress enabled coordinated migration of cancer cells by enhancing cell–ECM adhesion, suggesting that compressive stress can select for metastatic cell populations and trigger cancer cell invasion through surrounding tissues (55).

#### ECM-Induced Stress and ECM-Related Models

##### Culturing cells embedded in 3D collagen gels of varying concentration or degree of crosslinking

Several studies have used the *in vitro* model of culturing cells in 3D collagen gels (127–129). This system has showed that normal cell morphogenesis can be “tuned” by the balance between cell-generated contractility and opposing matrix stiffness (127). In addition, it has also been shown to facilitate the development of *in vitro* 3D bioengineered tumors that recapitulate the pre-vascularized stages of *in vivo* solid tumor progression (128). Modifying the collagen concentration, cell–ECM forces can be performed with a denser and, thus, stiffer matrix to produce larger forces. Using this approach, cancer cells are embedded in the ECM and direct cell–ECM interactions are measured.

Another way to modulate the forces around cancer cells without external application of mechanical stress is through the induction of increased ECM stiffness by LOX-mediated collagen crosslinking. In fact, Levental et al. showed that inducing collagen crosslinking stiffened the ECM, promoted focal adhesions, and induced the invasion of an oncogene-initiated epithelium, clearly suggesting that collagen crosslinking can regulate tissue stiffness to force focal adhesion formation and breast tumor malignancy (130).

##### Culturing cells in a 3D “sandwich” collagen gel set up

Another interesting approach that greatly mimics the tumor environment allowing rigorous control of various conditions was described by Brekhman and Neufeld in 2009. More specifically, they developed a novel asymmetric 3D *in vitro* culturing system and invasion assay by embedding a monolayer of tumor cells between two collagen layers. This assay was used to compare the invasive properties of several tumor cell types, therefore introducing a potent way to study the effects of tumor microenvironment on tumor cell invasiveness (131).

##### Culturing cells in 3D hyaluronic acid gels

Apart from collagen-based 3D gel culture systems, the use of hyaluronic acid-based 3D gels is increasingly gaining ground in cancer research in an attempt to better recapitulated normal tumor growth (132–134). Evidently, the practice of combining the two matrices is also being employed (134) by many researchers as a more physiologically relevant model, recapitulating the mixture of matrix components present in the tumor microenvironment (135).

##### Forming spheroids embedded in fibrous gels of increasing stiffness

In another method, tumor cells were embedded as spheroids in gels of varying agarose concentration, ranging from 0.3 to 2%, and the ability to form tumor spheroids was assessed by comparing spheroid formation in free suspension, as control (136). The study showed that colon carcinoma cells can form spheroids with a maximum diameter of 400 μm in 0.5% (w/v) agarose, while the spheroid diameter barely reached 50 μm in 1.0% agarose. Moreover, increased agarose concentration decreased cell proliferation in spheroids which was completely reversible once spheroids were removed from the gel (136). Similar studies have been also repeated with glioma, sarcoma, and breast cancer cells using either agarose or collagen matrix which further confirmed this inverse relationship between matrix stiffness and cancer cell proliferation (52, 137).

Interestingly, a combination of using a compression device and cell culture in agarose gels has also been used to assess the effect of external compression on tumor growth and metastasis using the “hanging droplet method” in order to generate cancer cell spheroids that later were incorporated into agarose gels (53). According to the “hanging droplet method,” cell suspension droplets were placed on the reverse of the cover of a culture dish and left in the incubator for 3 days (138). Subsequently, formed spheroids in hanging droplets were collected and re-suspended in 1.0% agarose solution and placed in inserts with porous membranes (0.4 mm in size). After solidification, the spheroid-gel construct was compressed using a piston of desired weight. The results of the study indicated that compressive stresses can suppress cancer cell proliferation and induce apoptotic cell death via the mitochondrial pathway (53).

##### Co-culture model systems

As described earlier, the tumor microenvironment consists of carcinoma cells, stromal cells, and intratumoral stroma, thus containing many cell types that are implicated in cancer development and progression. The development of co-culture model systems is an *in vitro* approach to study cell–cell communications or interactions among these cells that are thought to play an important role in cancer initiation, promotion, and progression (139, 140). This system offers a clear advantage compared to mono-culture systems that cannot evaluate interactions between carcinoma and intratumoral stromal cells.

##### Fibrous scaffold systems

Finally, fibrous scafford systems have also been developed recently. Of interest is a recent study where they produced a fibrous scaffold by electrospinning a mixture of poly(lactic-co-glycolic acid) and a block co-polymer of polylactic acid and mono-methoxypolyethylene glycol (141). The study showed that cancer cells cultured on this scaffold formed tight irregular aggregates similar to *in vivo* tumors that depended on the topography and net charge of the scaffold and the scaffolds induced tumor cells to undergo the epithelial-to-mesenchymal transition (141, 142).

Each and every one of the above-described approaches offers definite advantages and studies tumor growth from a different perspective while also presenting certain limitations. The methods using externally applied compressive stress can provide more precise information regarding the exact magnitude of the applied force whereas all the other models that generate stress by matrix stiffening cannot. On the other hand, modulating matrix stiffening is more physiologically and clinically relevant compared to externally applied mechanical forces. This is due to the fact that matrix stiffening methods maintain the structural architecture necessary for proper cell–cell and cell–ECM interactions and are therefore able to better recapitulating an *in vivo* phenotype and the true tumor microenvironment. In fact, several *in vitro* 3D models have been proposed to have the ability to acquire phenotypes and respond to different stimuli, thus bearing strong similarities to *in vivo* biological systems (128, 143–145).

However, most *in vitro* studies performed in the field of cancer biology have mostly relied on 2D cell culture studies. In that respect, atomic force microscopy (AFM) has been recently used as an important tool to visualize and quantify mechanical forces in cell culture systems.

### Atomic force microscopy in cell culture

Atomic force microscopy is a microscope that can be used to obtain images and other qualitative and quantitative information, such as mechanical properties, in a non-destructive manner, from a wide range of samples, including biological ones (146–149). AFM operates by measuring forces between a probe and the sample and offers extremely high resolution at the nanometer level, without special treatment, such as dehydration, labeling or coating, or vacuum conditions of the specimen (150, 151). Moreover, it operates well in samples embedded in water or buffers as well as on live cells; even detecting molecules at the single-molecule level. State-of-the-art AFM modalities, methods, and techniques can combine qualitative and quantitative information, such as high-resolution imaging with elasticity, modulus, adhesion, and deformation data, converting AFM into a powerful tool for researchers (150, 152).

Particularly, the use of AFM techniques in the field of cancer research was initially focused on the *in vitro* investigation of cancer cells. However, recent related techniques have begun to emerge as novel methodologies for unraveling “secrets” of the tumor microenvironment. The provided information ranges from morphometric imaging to force measurements and cover essential aspects of tumor microenvironment research. Nanoscale characteristics of cancer and stromal cells surface or pericellular alterations/activity can be provided, while nano-mechanical characteristics, like cells softness, ECM stiffness, and cell to ECM forces, can be acquired under almost physiological conditions.

In the literature, AFM has been used for:

a) Studying the effect of specific matrix remodeling molecules, such as TGFβ. AFM can be used to study biomechanical properties, such as cell stiffness, surface membrane features, elongation of cells, and interaction strengths during the EMT process, in response to TGFβ (153). With regard to the latter, AFM studies have shown an increase in the tension within the membrane after EMT induction (154), while alterations of cell topography and the formation of nodular protrusions at intercellular junctions were also demonstrated (155–157). In addition, increased cancer cell stiffness during EMT was also found to be a consequence of stress fiber formation and force generation.
b) Studying cell migration, invasion, and pericellular proteolytic activity as well as visualization/characterization of cell–ECM adhesions. Few research groups have used AFM to study invadopodia, the characteristic protrusions extended by tumor cells during invasion of neighboring tissue (158, 159). Moreover, AFM can contribute to the study of pericellular proteolytic activity of cancer cells since it can detect differences in the average height, volume, and molecular weight distribution of pericellular matrix proteins within the tumor microenvironment (160–162). AFM has also been used for the characterization of dual mechanical properties in prostate and breast cancer cells. In these studies, the investigation of both the cell–ECM and cell–cell adhesions showed that mechanical compliance alone fails to serve as a universal indicator for metastatic progression and, therefore, different therapeutic approaches should be considered for each tumor type in order to prevent metastasis in cancer patients (163). Finally, AFM has been used to determine the adhesion strength between an endothelial cell monolayer and tumor cells with different metastatic potential (164).

## Conclusion

Recent studies in the field of cancer research have shed light upon the critical role of tumor microenvironment for cancer progression, highlighted that understanding the interplay between cancer cells and their microenvironment can promote cancer pathogenesis and facilitate the development of more effective therapeutic approaches. The tumor microenvironment consists of tumor blood and lymphatic vessels and the tumor stroma. The latter contains non-cancer cells and tumor ECM components and its effects on cancer cell properties are considered pleiotropic. However, apart from regulating cancer cell behavior, abnormalities of the tumor vasculature and stroma pose barriers to the effective delivery of therapeutic agents, which can compromise treatment outcomes. Thus, understanding the tumor microenvironment and its abnormalities during cancer progression is fundamental for the development of better treatment strategies.

In this review article, we have summarized the common abnormalities observed in the tumor microenvironment, including tumor blood vessel hyperpermeability, and compression of intratumoral blood vessels due to the development of mechanical forces, as a result of stromal aberrations. We have also indicated molecules that could be used as targets in order to modulate tumor microenvironment, including angiogenic factors, such as VEGF, as well as ECM-remodeling growth factors, such as TGFβ (6). The ultimate research goal would be to make the tumor microenvironment phenotype less “cancerous” and more “normal” by targeting these molecules (165). Strategies that have been developed to normalize cancers include vessel normalization and stress alleviation techniques that can be used alone or in combination depending on tumor type (86).

Furthermore, we summarized the literature on available *in vitro* models used to study the tumor microenvironment that also take into account mechanical interactions between cancer cells and the tumor microenvironment. In general, *in vitro* models can be divided in those where stress is applied externally to cancer cells using a compression device and models where stress is induced by modulating the stiffness of the surrounding matrix increasing collagen content and/or degree of crosslinking. Last but not least, this review presents the advantages of using AFM as a novel technique to obtain qualitative and quantitative information on mechanical properties and nano-imaging of cells *in vitro* and in relation to matrix remodeling and cell–ECM interactions.

Finally, this review summarizes new considerations for the use of treatments that modify the tumor microenvironment. Desmoplastic tumors (e.g., pancreatic cancers, subsets of breast tumors) must experience high mechanical forces owing to the large amount of ECM and thus they should have a large amount of compressed vessels. In these tumor types, anti-VEGF drugs that fortify the vessel wall most likely would not work because the vessels will still remain compressed and dysfunctional and, thus, stress alleviation drugs to decompress vessels along with cytotoxic agents should be considered. Less desmoplastic tumors (e.g., subset of glioblastomas) are expected to have uncompressed vessels and in the case that these tumors are hyperpermeable, the use of anti-VEGF treatment with cytotoxic agents should be the therapeutic strategy of choice. In practice, one needs to identify which tumors have hyperpermeable vessels, compressed vessels, or both. This is a challenging task because although some broad statements can be made (e.g., PDACs are desmoplastic), there are many tumors where the degree of desmoplasia is highly variable from one tumor to the next, for the same tumor through time and potentially from the primary tumor to its metastases. To choose an appropriate strategy, the state of that individual tumor has to be known. Further development of imaging approaches and biomarkers should have the potential to help in this selection. Of course many other issues need still to be addressed particularly in order to identify novel therapeutic targets of the tumor microenvironment and better-tolerated pharmaceutical agents to complement and improve current therapeutic schemes.

## Conflict of Interest Statement

The authors declare that the research was conducted in the absence of any commercial or financial relationships that could be construed as a potential conflict of interest.

## Abbreviations

2D, two-dimensional; 3D, three dimensional; ACEs, angiotensin receptor enzymes; AFM, atomic force microscopy; Ang1/2, angiopoietin-1/2; ARBs, angiotensin receptor blockers; a-SMA, a-smooth muscle actin; CAFs, cancer-associated fibroblasts; CTGF, connective tissue growth factor; ECM, extracellular matrix; EMT, epithelial-to-mesenchymal transition; FG, fibroblast growth factor; GAG, glycosaminoglycan; HIF, hypoxia-inducible factor; LOX, lysyl oxidase; MMPs, matrix metalloproteases; MP, multiphoton; MSCs, mesenchymal stem cells; PBS, phosphate buffered saline; PDACs, pancreatic ductal adenocarcinomas; PDGF, platelet-derived growth factor; PGs, proteoglycans; POSTN, periostin; PHD1–3, prolyl hydroxylase domain proteins 1–3; RGS5, regulator of G-protein signaling 5; SDF-1, stromal cell-derived factor 1; SLRPs, small leucine-rich proteoglycans; TGFβ, transforming growth factor beta; VEGF, vascular endothelial growth factor.
